# Persistence selection between simulated biogeochemical cycle variants for their distinct effects on the Earth system

**DOI:** 10.1073/pnas.2406344122

**Published:** 2025-02-12

**Authors:** Richard A. Boyle, Edmund R. R. Moody, Gunnar Babcock, Daniel W. McShea, Sandra Álvarez-Carretero, Timothy M. Lenton, Philip C. J. Donoghue

**Affiliations:** ^a^Global Systems Institute, Faculty of Environment, Science and Economy, University of Exeter, Exeter EX4 4QE, United Kingdom; ^b^Bristol Paleobiology Group, School of Earth Sciences, University of Bristol, Bristol BS8 1TQ, United Kingdom; ^c^Department of Microbiology, College of Agricultural and Life sciences, Cornell University, Ithaca, NY 14853; ^d^Biology Department, Duke University, Durham, NC 27708

**Keywords:** Gaia hypothesis, persistence selection, its-the-song-not-the-singer theory, field theory

## Abstract

The average long-term impact of Darwinian evolution on Earth’s habitability remains extremely uncertain. Recent attempts to reconcile this uncertainty by “Darwinizing” nonreplicating biogeochemical processes subject to persistence-based selection conform with the historicity of the geochemical record but lack mechanistic clarity. Here, we present a theoretical framework showing how: 1) A biogeochemical “cycle-biota-variant” (CBV) can be defined non-arbitrarily as one biologically facilitated pathway for net recycling of an essential element, plus the genotypes driving the relevant interconversion reactions. 2) Distinct CBVs can be individuated if they have climatic or geochemical side effects that feed-back on relative persistence. 3) The separation of spatial/temporal scales between the dynamics of such effects and those of conventional Darwinian evolution can introduce a degree of randomness into the relationship between CBVs and their Earth system impact properties, loosely analogous to that between the biochemical causes and evolutionary effects of genetic mutation. 4) Threshold behavior in climate feedback can accentuate biotic impacts and lead to CBV-level “competitive exclusion”. 5) CBV-level persistence selection is observationally distinguishable from genotype-level selection by strong covariance between “internal” CBV properties (genotypes and reactions) and “external” climatic effects, which we argue is analogous to the covariance between fitness and traits under conventional Darwinian selection. These factors cannot circumvent the basic fact that local natural selection will often favor phenotypes that ultimately destabilize large-scale geochemical/climatic properties. However, we claim that our results nevertheless demonstrate the theoretical coherence of persistence-selection between non-replicating life–environment interaction patterns and therefore have broad biogeochemical applicability.

## Significance

Darwinian evolution is life’s fundamental sense-making principle and Earth is the only (known) planetary/climatic system capable of supporting life. However, the question of whether Darwinian evolution tends to improve or degrade planetary habitability has been contentious for decades. This is because natural selection is a population-level process that is spatially and temporally separated from the planetary-climatic scales at which habitability can be quantified. Here, we show theoretically how distinct variants of biogeochemical cycles can “compete” by climatic impact “phenotypes,” the effect of which is potentially rendered irreversible by geochemical feedbacks. This “Darwinized” way of looking at biogeochemistry may provide a framework for determining the effect of natural selection on habitability.

The sustained manifestation of habitable climatic conditions over Earth’s history is qualitatively distinct from comparable lifeless planets, such as Mars or Venus (1). A characteristic feature of habitable conditions is the planetary-scale atmospheric chemical disequilibrium that inspired James Lovelock to propose that life is an inherently planetary phenomenon (2, 3). This proposition has profound implications for any search for extraterrestrial life (4), as well as more basic existential significance, and remains broadly consistent with the evidence.

For example, the byproducts of life give rise to powerful biogeochemical recycling of essential nutrients (such as N, P, and S) at a global scale (5), such that the availability of biologically essential elements vastly exceeds that expected solely from abiotic influxes (6, 7). Furthermore, some statistical analyses suggest a topological similarity between the dynamics of climatic time-series data and the output of cybernetic control systems that are sensitive to not only the controlled variable, but also its integral and derivative (8, 9). On the other hand, it is also arguable that complex life has regularly undermined the habitable conditions initially allowing it to flourish (10) and that biology destabilizes its environment at least as frequently as it induces any form of homeostasis within it (11). This wide range of legitimate interpretations illustrates how a fundamental question remains unanswered: Does Earth support life because the Earth system sustains habitable conditions, or is the sustenance of habitable conditions causally connected to the presence of life?

The most significant barrier to answering the above question stems from a fundamental separation of scales between climatic/geochemical and Darwinian dynamics. The “Life” relevant to planetary scale climatic habitability is a planetary scale entity; the totality of the biota (12). By contrast, natural selection acts upon the differential survival and reproduction of interacting organisms within biological populations (13), in a manner that is locally contextualized and causally decoupled from any long-term consequences. Some would argue that the legitimate response to this separation is simply to accept that habitability must be framed in terms of other concepts, such as maximum entropy production (14), anthropic observer bias (15), the balance between luck and deterministic factors (16), and physical constraints on evolutionary adaptation (17). In this light, Darwinian evolution within biological populations can arguably be subsumed into the totality of global-scale feedback processes (18) from which habitability emerges.

While they provide informative explanations for numerous observations, such approaches leave Darwinism-centric questions unanswered. For instance, if planetary habitability is an unselected byproduct of life’s presence (as opposed to anything even loosely analogous to an adaptation), why are the byproducts of abiotic processes not conducive to the emergence of homeostasis, and what exactly explains the *difference* between Earth and similar lifeless planets?

The separation between Darwinian and planetary-climatic scales is the foundation of Ford Doolittle’s recent “it’s-the-song-not-the-singers” (ITSNTS) theory, which attempts to derive a “Darwinized Gaia” (19), based on a relaxed concept of natural selection applicable to nonreplicating entities, including global biogeochemical cycles (20). ITSNTS revisits Doolittle’s initial staunch and explicitly Darwinian criticism (21) of Lovelock’s Gaia hypothesis. The latter proposes the existence of an emergent planetary scale homeostatic system, that provides a legitimate basis for life’s maintaining Earth in a state that is “good for life” (22). The (notoriously severe) Darwinian criticism of Gaia focused on how neither Earth, nor any planetary scale entity influencing habitability, exists in an interacting population of comparable variants, and so no such entity can experience natural selection. Given that natural selection is biology’s principal sense-maker, the whole proposition is still typically viewed by evolutionary biologists as dubious at best.

ITSNTS attempts to mitigate this concern by expanding the concept of selection beyond its conventional bounds, focusing on subplanetary but near-global scale processes coevolving with the collective sets of genotypes that sustain them. The crux of ITSNTS is the concept of “recruitment,” by biogeochemical cycles, of the sets of genotypes that interconvert distinct species of the relevant element/nutrient (nitrate and ammonium, sulfide and sulfate, etc.) (23). Continuous reassociation between such genotypes and the nutrients/growth substrates on which they depend constitutes “re-production” (i.e. continuous regeneration) of the cycle-biota interaction, such that this interaction has something akin to a fitness of its own. Crucially, ITSNTS suggests that this makes a difference to biological allele frequencies: Novel alleles/genotypes arise and increase from rarity via natural selection by means of an interaction with a geochemical cycle subject to persistence-selection, where without this interaction such biological novelties would not arise, because the relevant taxa would be extinct or fundamentally different.

However, any biogeochemical cycle can immediately be said to be “fundamentally different” from a hypothetical alternative version of itself not subject to equivalent geochemical boundary conditions. Testing this kind of biogeochemical historicity is just as challenging as investigating the balance between chance and necessity within Darwinian evolution by “replaying life’s tape” (24). At this point, it therefore becomes useful to define a suitable null hypothesis, as the default view to which ITSNTS can be compared. On what is perhaps best-termed the “byproducts plus feedback” (BPF) view, biogeochemical cycles exist at the planetary scale simply because living things release physiological byproducts into the environment: Geochemical boundary conditions may affect which metabolisms are feasible in a particular context, but it is unparsimonious and unnecessary to invoke persistence-selection at the level of cycle biota interactions.

In this work, we aim to provide a theoretical framework that begins to distinguish between the likely predictions of these two views.

## Conceptual Suggestions and Model Results

**Fig. 1.**
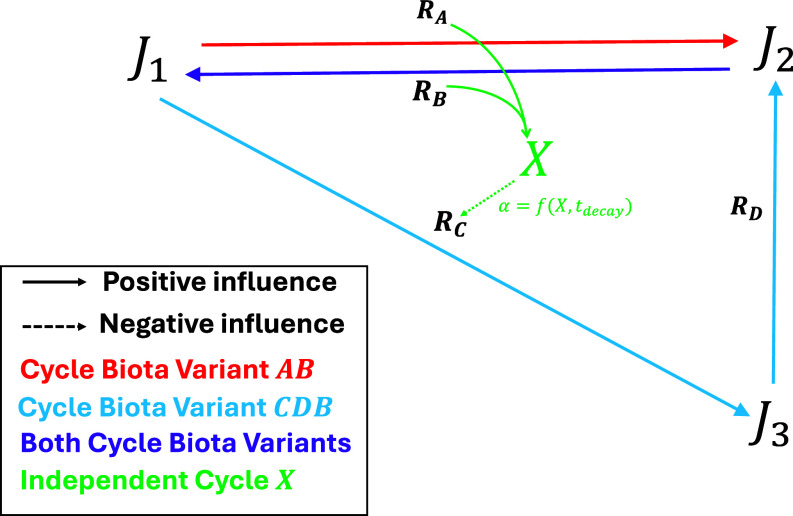
The general concept of cycle individualization by external impact on biogeochemical processes. A generic biologically essential element *J* is interconverted between three distinct variants *J*_1_, *J*_2_, *J*_3_ as a physiological byproduct of biological “reactions” *R_A_*, *R_B_, R_C_*, *R_D_* (solid arrows depict increasing influence). An intuitive definition of a putatively persistence-selected CBV as one pathway for net recycling of *J* leads to two distinct CBVs being identifiable, *AB* (red) and *CDB* (blue), but also illustrates how process-level interconnectedness (purple, reaction *R_B_*) may render such a definition arbitrary. This is less of a problem given the additional assumption that the combined byproducts of reactions *R_A_* and *R_B_* impact upon a different geoclimatic system *X*, ultimately feeding back negatively (dashed arrows) on reaction *R_C_*, therefore influencing on CBV relative persistence.

Consider Fig. 1, which depicts (what we argue is) the minimal core structure of a biogeochemical cycle. The system focuses on an idealized biologically essential element *J*, occurring in three interconvertible subvariants *J*_1_, *J*_2_, *J*_3_, within a well-mixed and materially open system, roughly analogous to the marine pool of a key nutrient (see *Materials and Methods* for full model description). Each “*J*-variant” is subject to abiotic fluxes through which it enters the system from the outside (analogous to weathering or tectonically driven inputs) and is removed from the system in linear proportion to its level (analogous to abiotic burial fluxes). Each *J*-variant is also subject to biologically driven interconversion reactions. The system tracks the biomass and genetic composition of four distinct species *S_A_*, *S_B_*, *S_C_*, and *S_D_*—each comprising two genotypes, the *p* genotype, which performs the relevant *J*-variant interconversion, and the *q* genotype, which does not. Each reaction rate thus varies linearly with the number of *p* genotype individuals in the relevant species. The relative fitness of each genotype within each species is a function of an explicit selection parameter, and mutation between the two genotypes occurs at reproduction at a parameterized probability.

If ITSNTS-style persistence selection is to be taken seriously, it must differentially act on a certain kind of entity. In the above context, an obvious possibility would be to define a cycle-biota-variant (CBV) as a single path for net recycling of *J* (or an equivalent real biologically essential nutrient), including the relevant alleles. Global scale metrics like the cycling ratio (between the quantity of an element within the biosphere and the influx from the outside (7)) might legitimize such a definition. But (as Fig. 1 shows) there exist two distinct but overlapping paths for net recycling of *J*, reaction sequences *A*→*B* and *C*→*D*→*B*. Is this two CBVs, or one CBV with some internal structure? Even at this bare minimum level of complexity, it is not obvious that there exists a nonarbitrary answer to this question. This problem of definitional arbitrariness will only get worse on scaling up to real examples, given the highly materially interconnected nature of biogeochemical cycles. This, we suggest, is an irreconcilable problem for any putative process-level persistence selection—if differentially persisting cycles are defined solely in terms of their internal material/energetic properties.

But Fig. 1 also depicts the idea that the reactions *A* and *B* interact with each other, via an additional idealized climatic variable *X*. Specifically, the combined byproducts of reactions *A* and *B* produce byproduct flux $X_{\mathrm{var}}=f\left(A∙B\right)$. This flux reacts with a partially isolated subreservoir within the “*X*-cycle,” triggering a feedback sequence that ultimately increases *X*. Where this increase exceeds a growth inhibition threshold, the result is a suppression function α=f(X) that reduces the biomass of species *C*, therefore the corresponding reaction and CBV, giving the following causal sequence:

$${\mathrm{CBV}}_{\mathrm{AB}}\overset{\mathrm{increases}}{\to }\alpha (X)\overset{\mathrm{decreases}}{\to }S_{C}\overset{\mathrm{increases}}{\to }\frac{{\mathrm{CBV}}_{\mathrm{AB}}}{{\mathrm{CBV}}_{\mathrm{AB}}+{\mathrm{CBV}}_{\mathrm{CDB}}}.$$

Thus, we have an idealized model of biologically driven nutrient recycling, which potentially has climatic side effects that feed-back upon this recycling and the relevant alleles. A niche constructionist perspective (25), whereby genotypes alter and are altered by their environmental states, with a knock-on effect on fitness, could arguably deal with this scenario straightforwardly, as could various models of interference competition in evolutionary ecology. Is there any need to invoke ITSNTS-style, cycle-level persistence selection on making the jump to geochemical scales?

We suggest that biological impact on global geochemical and climatic properties potentially differs from more localized effects in terms of potential irreversibility, and degree of causal decoupling from the biology itself. Both these factors are ultimately due to a separation of spatial and temporal scales. In conventional evolutionary ecology, resource-based interference competition often results in density-dependent priority effects (26) rather than necessarily leading to extinction. While autocatalytic interactions are commonplace in ecology (27), the idea of individuation of different autocatalytic loops by their effect on background conditions is likely implausible unless such effects are irreversible. By contrast, if one component of the biosphere has a global climatic effect, which triggers an irreversible change in the affected variable via feedbacks intrinsic to that variable, it becomes more realistic that initial allele frequencies will be altered in a similarly irreversible way. The climatic factors dictating whether this does happen will be causally decoupled from (i.e. random with respect to) the biological factors dictating the nature of the initial effect. We propose that this partial separability of global biogeochemical dynamics from those of Darwinian evolution, combined with the potential for irreversible directional changes, may potentially individuate distinct CBVs, providing variation upon which persistence selection acts.

**Fig. 2.**
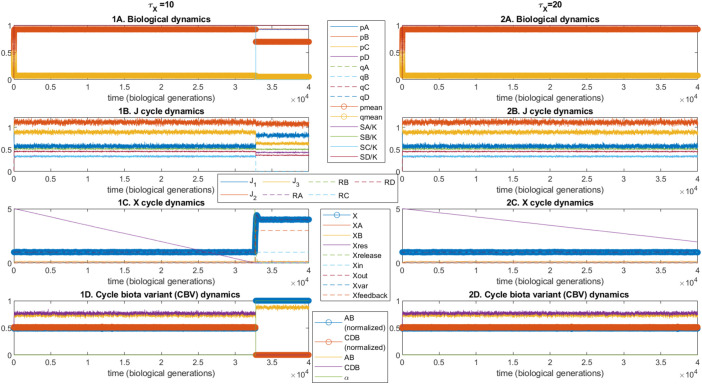
A simple timescale separation is sufficient for CBV-level dynamics to make a difference to gene frequencies. The impact of the timescale separation $\tau_{X}$ between the *X*-cycle (row C) and the biological (row A) and *J*-cycle (row B) dynamics, upon two single-simulation results (column 1, $\tau_{X}=10$, and column 2, $\tau_{X}=20$) for otherwise identical parameter choices. With sufficient timescale overlap (column 1), the byproduct flux from species A and B reacts with the $X_{\mathrm{res}}$ reservoir, until its depletion leads to a discontinuity in the *X*-cycle (1C), the subsequent state of which suppresses the growth of species C, leading to the displacement of CBV *CDB* by *AB* (1D). This change is associated with a decrease in the cross-species mean frequency of the reaction-performing *p*-allele (1A) but fixation of *p_A_* and *p_B_*. A minor increase in the timescale separation (column 2) prevents any change from the initial equilibrium state.

These issues are represented in our model by a parameterized timescale separation between different sets of dynamics, some of which exhibit the potential for positive feedbacks distinct from the biology. The way in which the suppression effect *α*(*X*) manifests is a function of the timestep relevant to the *X*-cycle, which (by hypothesis) is longer than those of the biological dynamics by a parameterized factor $\tau_{X}$. Fig. 2 compares numerical integrations of the full system of stochastic differential equations (methods) for different values of this timescale separation parameter $\tau_{X}$. The threshold behavior arises from the fact that the biological byproduct flux $X_{\mathrm{var}}=f\left(A∙B\right)$ reacts with a partially isolated subreservoir within the *X* cycle, which is not released into the system until the entire reservoir has undergone this reaction (analogous to, for example, the impact on temperature or atmospheric composition of the sudden release of an initially frozen reservoir of greenhouse gas). The release of this reservoir triggers a discontinuity due to feedback built into the *X* cycle and “switches on” the suppression function in a relatively discrete way. Whether or not this sequence occurs depends upon a fixed number of units of *X*, which must be “titrated” by the byproduct flux, and the rate of this byproduct reaction per biological generation is reduced when the timescale separation $\tau_{X}$ is greater. Unsurprisingly therefore, when otherwise identical dynamics are “spread” over a greater number of biological generations (by increasing $\tau_{X}$) the solution is qualitatively altered. While in terms of the model implementation, the explanation for this difference is trivial, we suggest that conceptually it illustrates the above point that the timescales over which climatic and geochemical variables adjust will, in many cases, be randomly distributed with respect to those of Darwinian evolution, providing an extra degree of freedom that may generate (or destroy) correlations between biological byproducts and their wider climatic consequences.

**Fig. 3.**
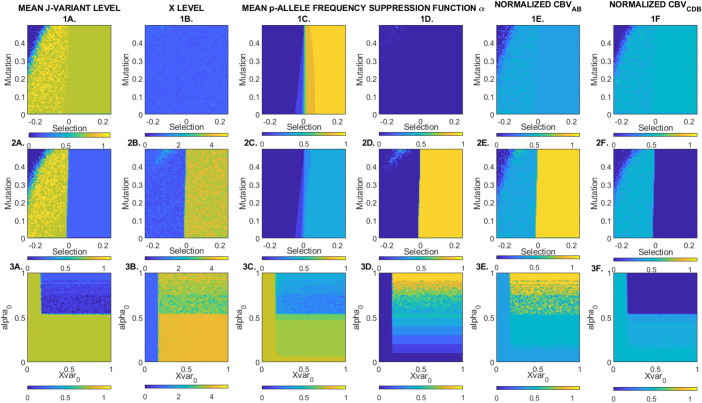
Replicate-averaged steady state solutions of the biogeochemical mode under different boundary conditions. Each color point shows the average of 1,000 simulations equivalent to those given in Fig. 2. The *Top* row shows the effect of changing the baseline selection and mutation parameters, first under boundary conditions in which the biological byproduct flux is always zero (there is no feedback via the X cycle, $X_{var0}=0$), the second row under conditions in which it is an increasing function of the product of reactions A and B (this feedback does exist, $X_{var0}=1$). The *Bottom* row shows (for constant selection and mutation coefficients) the effect of incremental variation in the baseline magnitude of the byproduct flux $X_{var0}$ and the suppression function $\alpha_{0}$. Columns A-F show, respectively, the mean level of *J*-variant (i.e., $\frac{1}{3}\left(J_{1}+J_{2}+J_{3}\right)$), the value of *X*, the mean (cross species averaged) relative frequency of the reaction-performing *p*-genotype, the final value of the suppression function α=f(X), and the final normalized relative value of the two CBVs.

Fig. 3 shows replicate averaged steady state solutions of the biogeochemical model for different selection and mutation rates, and different susceptibilities to feedback with the *X*-cycle (the latter implemented by changing the baseline magnitude of the above-mentioned byproduct flux and suppression function). These results demonstrate a degree of decoupling between genotype-level selection in isolation (*Top* row), selection with the potential for feedback that alters the underlying steady states (*Middle* row), and changes in susceptibility to CBV-level persistence selection with constant Darwinian parameters (*Bottom* row). Maximal recycling of *J* occurs when all reaction-performing genotypes are strongly selected, without any *X*-cycle feedback. In this case, recycling is a byproduct of genotype-level selection (interpretable as BPF above). When the potential for the feedback is added, and the baseline magnitude of the byproduct flux and suppression function is high ($X_{var0}=1\alpha_{0}=1$, *Middle* row), selection for reaction-performing *p*-genotypes (which always occurs at a uniform strength across species) generally triggers displacement of ${\mathrm{CBV}}_{\mathrm{CDB}}$ by ${\mathrm{CBV}}_{\mathrm{AB}}$. The same change is observed as the values of these parameters are increased incrementally for constant selection and mutation rates. Note that extinction of the reaction-performing genotypes in ${\mathrm{CBV}}_{\mathrm{CDB}}$ leads to a decline in the mean value of *J*, a form of “tragedy of the commons” (28). This highlights how the form of CBV-based dynamics that we propose is, ultimately, a form of competition between emergent life–environment interaction patterns, as opposed to some constraint directed toward a planetary optimum state. Note also that boundary conditions supporting CBV-level competitive exclusion may be associated with strong selection in favor of the reaction-performing genotypes in ${\mathrm{CBV}}_{\mathrm{CDB}}$ that are ultimately driven extinct, by the compensating impact of the suppression function α=f(X). At no point is there differential genotype-level selection in favor of the reaction-performing alleles in one CBV over those in another.

**Fig. 4.**
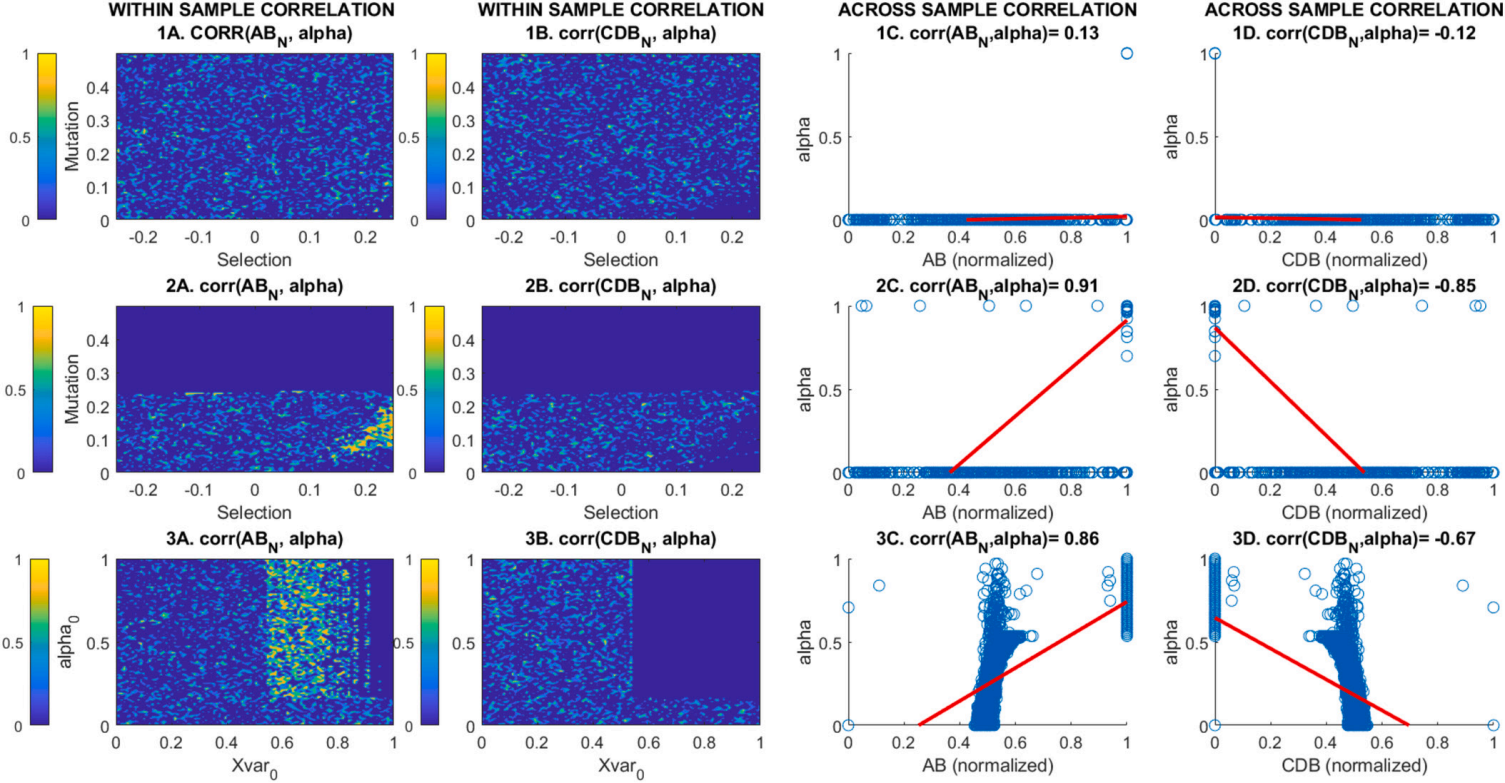
Correlations between steady state normalized CBVs and suppression function values across different boundary conditions. Rows correspond to those in Fig. 3, i.e., sensitivity analyses to selection and mutation with no *X* cycle feedback (row 1), selection and mutation with *X* cycle feedback (row 2), and baseline magnitudes of the byproduct flux $X_{var0}$ and suppression function $\alpha_{0}$ for constant selection and mutation rate (row 3). Within each row, the first two columns show the within-sample correlation—i.e., the correlation between the final values of the variables across the set of replicates for the parameter combination shown on the X and Y axes. The second two columns show a scatterplot of all final steady state values, with a 1st-degree polynomial linear regression line (red) giving the value of the correlation across the entire dataset.

Fig. 4 shows how the regions of parameter space that cause the system to exhibit the above behavior are characterized by strong positive covariance between the steady state value of this suppression function *α* and the direction in which persistence selection is occurring (i.e., in favor of ${\mathrm{CBV}}_{\mathrm{AB}}$ against ${\mathrm{CBV}}_{\mathrm{CDB}}$). Note that CBV-level competitive exclusion $\frac{{\mathrm{CBV}}_{\mathrm{AB}}}{{\mathrm{CBV}}_{\mathrm{AB}}+{\mathrm{CBV}}_{\mathrm{CDB}}}\to 1$ reliably tracks this covariance. The strength and direction of such covariance mirrors that of persistence selection itself and is not an artifact of genotype-level selection in favor of the reaction-performing alleles, because it is contingent upon the value of environmental parameters that are decoupled from the biology: The only parameters being changed on the *Bottom* row are those relevant to the *X*-cycle, which has no relevance to within-species relative fitness of any reaction-performing genotype. We suggest that this, coupled with the above-mentioned timescale separation, distinguishes this CBV-level persistence selection from a more typical Darwinian explanation in terms of “group-viability selection,” or equivalent. In other words, we argue that this ITSNTS-style persistence selection between CBVs amounts to group-viability selection, through randomly initiated covariances with climatic/geochemical byproducts that are potentially spatially and temporally decoupled from Darwinian dynamics.

Additionally, we direct the interested reader to results from a supplementary model (*SI Appendix*, *Supplementary methods* S1 and Figs. S1 and S2) from which we estimate the probability that dynamics of this sort may occur, by partitioning the model structure into the following contingent events:

1. A synergistic/symbiotic life–environment interaction arises via an environmental state, with dynamics partially overlapping with those of Darwinian evolution.
2. This interaction persists despite the possibility that local natural selection may undermine it.
3. This persistence lasts long enough for a side effect, or geochemical/climatic “phenotype,” of the interaction to manifest, over the relevant larger/longer scales.
4. This global geoclimatic side effect feeds back on local evolutionary ecology to modify the stability and relative persistence of this interaction (most realistically by killing-off competitors).

*SI Appendix*, Figs. S1 and S2 compare sampling-based and analytic estimates of this probability, relative to parameters, including the number of states in the local and global environmental contexts, and various Darwinian terms such as the average selection coefficient and the effective population size. Our best guess at the probability of the combined occurrence of the above events is roughly ∼1/20, that is low but nonnegligible (typically being maximized in small populations, situations in which conventional Darwinian dynamics operate in the same direction as CBV-persistence-selection, and cases in which the overall state space is small). This low probability is, of course, more compatible with the involvement of persistence selection in rare ecophysiological reorganizations than incremental microevolution.

Finally, we summarize existing phylogenetic work illustrating the interactivity between climatic context and the taxonomic distribution of physiological diversity. *SI Appendix*, *Supplementary methods* section S2 and associated (*SI Appendix*, Figs. S3 and S4) show phylogenetic reconstructions of the “completeness” of the metabolic pathways contained with different global biogeochemical cycles across time, relative to the modern state. Using a recently published dataset (29) we compare how the completeness of nitrogen and methane metabolic pathways have been affected over time, and we see a similar downward trend in methane-related metabolic pathways until after 2Ga, as well as a corresponding upward trend in nitrogen fixation and nitrate reduction pathways. While this pattern is inherently an increasing one, the rate of increase is nonuniform, and can be related to major changes in environmental boundary conditions, most obviously the 2.3 Ga Great Oxidation Event (GOE), which had a profound impact on almost all prokaryotic metabolisms around that time, reducing anaerobic metabolisms such as photoferrotrophy and methanogenesis (30), and increasing aerobic metabolisms (31).

## Discussion

Our results provide, we argue, a theoretical framework showing how persistence-based selection can act on subvariants (CBVs) of global biogeochemical cycles, if they are individualized by their impact on a wider geoclimatic context. This supports Doolittle’s ITSNTS theory (23) and a recent philosophical formalization of Darwinian covariance relationships in context of the Gaia hypothesis (32). Furthermore, we suggest that our theoretical results sketch a pattern that has been common during Earth history: displacement of distinct life–environment interaction patterns via changes in wider boundary conditions, often mediated by life itself, impacting upon taxonomic diversity and the relative abundance/growth of distinct physiologies.

### Examples from Earth history.

The evolution of oxygenic photosynthesis resulted in the displacement of previous anoxygenic variants, by removing dependency on a geochemical supply of electron sources, and enhancing free energy capture via the fusion of two photosystems (33). The production of free oxygen culminated in the GOE: oxidation of Earth’s atmosphere, oceans, and land surface, which constitutes the single most significant geochemical change in the planet’s history (34). The widespread exploitation of the increased respiratory free energy yield resulting from the use of oxygen as an electron acceptor allowed the explosive proliferation of aerobic heterotrophy and a relative increase in extinction among many anaerobic lineages (31). If we interpret, in a loose sense, the coupling between oxygenic photosynthesis and aerobic respiration, as one “CBV,” and that between anoxygenic photosynthesis reactions and the use of anaerobic electron acceptors as another (or a set of others), then it becomes clear that the oxygenic CBV had environmental effects (widespread oxidation of surface environments and influxes) that made most of the planet uninhabitable for the other.

Various other case studies fit this qualitative framework. Components of the sulfur cycle connected to sulfide oxidation underwent, following the GOE, taxonomic diversification facilitated by horizontal gene transfer and gene duplication (35). During the early stages of Earth’s oxidation, an oxygen-poor, Fe(II) rich state may conceivably have been created by anoxygenic phototrophs depriving cyanobacteria of light in the photic zone—creating an inertia that temporarily resisted oxidation of the surface ocean (36). The Proterozoic nitrogen cycle was likely dominated by a deep-water pool of ammonium prior to the build-up of a stable marine nitrate reservoir. This low-oxygen variant of the N-cycle may have constituted a net oxygen sink with respect to the ocean-atmosphere (connected to the combination of nitrification of ammonium, nitrate-driven primary production and significant respiratory denitrification under low oxygen boundary conditions, collectively consuming more oxygen than that produced by the corresponding photosynthetic primary production (37)). Thus, this variant conceivably “suppressed” the rise of a competitor (the modern, high oxygen N-cycle characterized by a larger baseline nitrate pool (38)).

Finally, many metabolism-first origin of life models continue to focus on autocatalytic networks of chemical reactions (39) existing prior to the genetic apparatus for biological information propagation. If two distinct autocatalytic chemical networks can be conceived of as competing via byproduct-driven knock-on effects on some environmental context, then the winner of this competition may be more likely to persist long enough to become coupled to protocells, nucleic acid, and other prerequisites for abiogenesis (40).

### Theoretical Considerations.

A natural overlap presents itself between our arguments and ideas that show how selection can generate directional processes which are self-re-enforcing when they adhere to a certain trajectory. One such idea is field theory (41), which provides a naturalistic account of systems that exhibit persistence and plasticity (as they are defined in systems theory and cybernetics), holding to a certain trajectory in the face of perturbations (42). It sees certain persistent and plastic “movements” of a Darwinian unit of selection up a fitness landscape as being governed by the structure of an “ecological field” (43). Importantly, this line of reasoning is applicable at different spatial and temporal scales and embraces differential persistence. In these terms, distinct CBVs are locatable, by means of their geochemical/climatic impact, in “persistence fields” analogous to conventional fitness landscapes; the CBVs are individualized by means of the distinct trajectories that they adopt with respect to such fields.

The premise that natural selection can legitimately be said to act on nonreplicating entities creates a potential variation supply problem. Without the iterative occurrence of replication with mutation, the action of persistence selection is potentially restricted to stability-based sorting (44) within bounds defined by the variation initially present in the population of interest. For some, this renders persistence selection relatively uninteresting (45), although others question whether lineage formation is essential for Darwinian evolution to occur (46). To some extent, the original conception of ITSNTS attenuates this concern through “recruitment” of genotypes by cycles, in the sense of perpetuation of the relevant niches, and re-production (continuous regeneration) of cycle biota variants. Thus, the biosphere provides variation in the normal way, with the stipulation that a persistence-selected component of fitness, derived from the association with a global biogeochemical cycle, makes a difference to allele frequencies (23). In previous work, we have suggested that any such cycle-associated component of fitness may be particularly relevant to net genetic assimilation of ancestrally facultative physiological traits (47). Thus, in the original formulation of ITNTS, the differential persistence of the cycle as a whole constitutes an interactor (48) that affects the fitness of a group of replicators, with compatible niches and physiologies.

Here, we have suggested a clear potential definition of what such a persistence-selected-interactor amounts to and how it is distinguishable from competitors: A single pathway for net recycling of a biologically essential nutrient, plus the taxa driving the relevant interconversion reactions, coupled to a lasting effect on the Earth system that influences relative persistence. Recruitment is implicitly represented here by the combination of mutation from the *q* genotype to the *p* genotype, and the continuing availability of the relevant substrate nutrients, which creates a niche for the latter when the wider environmental context (the *X*-cycle) allows. Changes in this context (the suppression effect) alter the capacity of different CBVs to “re-produce.” Our model also shows how CBV impact “phenotypes” 1) can manifest at the level of the cycle as a whole (the impact on the *X*-cycle derives from the interaction between the byproducts of two biologically driven reactions), 2) may be contingent upon features of global geochemical cycles entirely uncorrelated with Darwinian dynamics (the change in relative timestep in Fig. 2 and 3) can have a potentially dramatic knock-on effect on relative persistence (the potential for positive feedback and threshold behavior within the *X*-cycle that drives CBV-level “competitive exclusion”).

The novelty-generating capacity of Darwinian evolution derives from the combination of the “randomness” of mutation and the filter-like impact of selection (49). The former property results from the fact that the probability of a given mutation’s occurrence is unaffected by the impact it will have on fitness if it does occur – which in turn derives from the decoupling of the biochemical causes of a mutation from its phenotypic and evolutionary consequences (50, 51). We propose a loose analogy between this “randomness,” and the way in which the immediate evolutionary/ecological factors that cause a CBV to arise and spread are decoupled from the causes of any climatic impact this CBV may have (Table 2).

For instance, the climatic feedbacks that likely stabilized a high oxygen atmosphere (52) were entirely independent from the selection pressures within the biosphere relevant to the origin and early evolution of oxygenic photosynthesis. Nevertheless, these climatic feedbacks significantly impacted upon the subsequent evolution and genetic composition of the aerobic component of the biosphere, explaining their difference from taxa rendered extinct or marginalized by the GOE. By comparison, the kind of autocatalytic interactions represented here by CBVs also occur frequently in more localized ecological contexts (27), some of which may persist longer than others. But when the “persistence-field” comprises a localized variable continuously adjusting to biological growth over the same spatial and temporal scales, the sort of step-changes necessary to irreversibly alter allele frequencies are less likely (rather some kind of dynamic equilibrium involving density-dependent growth limitation will be more probable). In this vein, we stress that our supplementary model illustrates how this pattern of CBV-level persistence selection is likely restricted to relatively rare and major biogeochemical reorganizations.

Persistence selection also has implications for sequential selection (53): a cyclical process whereby the biosphere exerts a destabilizing influence that pushes the climate system to the bounds of habitability, which then leads to extinction, followed by biological recolonization resulting in a “reset” of the life–environment interaction (54). The idea is that multiple iterations of this sequence of events eventually lead to a self-stabilizing system (for example some argue that each reset increases the size of a reservoir of biodiversity, sufficient to increase the likelihood of a habitability promoting life–environment interaction (55)). The problem is the (debatable) assumption that no one reset of the planetary life–environment interaction results in irreversible loss of habitability (56), which arguably invites the question addressed by the Gaia hypothesis in the first place. CBV-level persistence selection constitutes a partial bridging of this separation of scales, by introducing a distinct level of adaptation, and a more bounded climatic impact that affects one type of life–environment interaction over another–which might allow for a more restricted form of sequential selection less likely to lead to complete biospheric extinction.

We stress that this theoretical framework should not be overinterpreted to conclude that all geochemical/climatic patterns are the product of persistence selection, or to disregard the basic constraint that a genotype with high relative fitness over short timescales will still be favored over such timescales, even if it ultimately drives itself (and/or the rest of the biosphere) extinct over longer timescales. The process we invoke here is ultimately one of niche-stabilization and/or destruction at global scales, likely restricted to major and revolutionary biogeochemical reorganizations—not anything analogous to the incremental production of complex adaptations in conventional Darwinian evolution. Nonetheless, the mechanism we suggest here constitutes a partial bridging of this separation of scales, introducing a distinct level of persistence-based “adaptation.” This reframing of Earth history in terms of competitive displacement of one CBV by another provides a perspective that allows for an authentically Darwinian (and indeed competition-centric) understanding of the emergence of a planetary scale feedback system, potentially warranting the label of “Darwinized Gaia.”

## Materials and Methods

Model 1, the system of stochastic differential equations corresponding to partially coupled Darwinian and geoclimatic dynamics (Figs. 2 and 3), is described here, with parameters given in Table 1. Model 2, a discretized formulation of the events in sample space necessary to generate the probabilities discussed, is described in the supplementary information. As discussed in the main text, the former dynamical model represents a materially/energetically open well-mixed system, consisting of three interconvertible variants (*J*_1_, *J*_2_, and *J*_3_) of a generic biologically essential element *J.*

**Table 1.** Default parameter choices for biogeochemical model

Parameter	Meaning	Default value
$F_{\mathrm{in}}$	Units of *J* entering system from the outside per timestep	$F_{in0}=1$
$F_{\mathrm{out}}$	Corresponding out flux	$F_{out0}=F_{in0}$
$R_{\max}$	Maximum interconversion reaction rate	$R_{\max}=F_{in0}$
*K*	Ecological carrying capacity for each species	*K* = 1,000
$\bar{r_{n}}$	Enhancement of interconversion reaction per biological individual	$\bar{r_{n}}=1/K$
$K_{m}$	Concentration/level of substrate *J*-variant at which reaction rate is half *R_max_*	*K_m_* = 1
$g_{0,i}$	Baseline effective population growth rate (i.e., births-deaths per unit time)	$g_{0,i}=1$
$W_{0}$	Baseline absolute fitness (average potential offspring per individual per time)	$W_{0}=1$
*s*	Sensitivity of the fitness of the reaction-performing *p* allele to the level of substrate *J*-variant	*s* = 0.01
$J_{i0}$	Baseline *J*-variant level for normalization	$J_{i0}=F_{in0}$
$m_{0}$	Mutation parameter, corresponding to the probability that a reproductive event will result in an offspring with a genotype different from that of the parent	$m_{0}=0.1$
$\varepsilon_{X}$	Fraction of *J*-element that is transferred to the *X*-cycle as a byproduct of the reactions in ${\mathrm{CBV}}_{\mathrm{AB}}$	$\varepsilon_{X}=0.5$
$X_{rel0}$	Baseline rate of release of *X* from transient reservoir $X_{res2}$ once reservoir $X_{\mathrm{res}}$ has been depleted to zero	$X_{rel0}=1$
$X_{feedback0}$	Baseline value in bistable feedback	$X_{feedback0}=3$
$X_{\mathrm{on}}$	Lower threshold for unstable region in *X* cycle	$X_{\mathrm{on}}=3$
$X_{\mathrm{off}}$	Upper threshold for unstable region in *X* cycle	$X_{\mathrm{off}}=6$
a,b	Parameters dictating shape of sigmoid in feedback flux	a=b=10
τ	Ratios between the timescales of the different sets of dynamics	τ≫1, model parameter
$X_{\mathrm{suppress}}$	Value of *X* at which suppression variable *α* becomes nonzero	$X_{\mathrm{suppress}}=3.5$, model parameter
$\alpha_{0}$	Baseline value of suppression term	$0<\alpha_{0}<1$, model parameter
$\bar{r_{n}}$	Increase in effective substrate availability per biological individual	$\bar{r_{n}}=\frac{1}{K}$
ω	Maximum bioavailable fraction of substrate *J*-variant pool	ω=1

**Table 2.** Suggested analogy between the geochemical impact of CBVs and the evolutionary impact of mutations

Category of influence	Conventional Darwinian evolution	ITSNTS by cycle competition
Level of causation 1: Source of variability	Biochemical and molecular biological causes of genetic mutation	Internal change in nutrient recycling loop topology, resulting in new CBV
Level of causation 2: Consequence of variability, affected by but causally decoupled from (1)	Impact of mutation on phenotype	External impact upon Earth-system feedback, particularly directional and/or irreversible geochemical changes
Basis for closure of feedback between levels of causation (2) and (1)	Impact of phenotypic change on evolutionary dynamics of genotype, affecting relative frequency of mutation	Directional changes in the totality of Earth-system feedbacks, with knock-on effect of feedbacks/changes on relative persistence of distinct CBVs
Overall implication	Indefinite potential for change in genotype/phenotype frequency by mutation plus selection	Long-term change in CBV relative persistence and topology of biogeochemical cycles

### Biological Dynamics.

The number of reaction-promoting individuals $n_{\mathrm{pi}}$ in species *i* is the product of the relative frequency of the *p*-genotype and the total number of individuals in the relevant species (rounded to the nearest integer):

[1] $$n_{\mathrm{pi}}=p_{i}∙M_{i}.$$

The total number of individuals in each species is a function of an effective population growth rate $g_{0,i}$ and a species-specific carrying capacity *K*:

[2] $$\frac{dM_{i}}{\mathrm{dt}}=g_{0,i}∙M_{i}∙{\left(1-\frac{M_{i}}{K}\right)}^{2}.$$

The absolute fitness of each genotype is the average number of offspring individuals produced per individual of that genotype per unit time. For the *q* genotype this is a constant, for the *p* genotype this constant is multiplied by the level of the substrate *J*-variant and a sensitivity constant *s*:

[3] $$W_{A(p_{i})}=W_{0}∙\left(1+s∙J_{x}\right),$$

[4] $$W_{A(q_{i})}=W_{0},$$

where again, *x* = 1, 2, 3 and i = *A*, *B*, *C*, *D* as appropriate. Thus, *s* = 0 represents a case in which the CBVs are entirely byproducts, *s* > 0 represents a case in which the presence of the substrate enhances the fitness of the genotype driving the reaction, *s* < 0 represents a case in which the *p* genotype self-limits by performing a reaction that undermines its own relative fitness advantage or experiences some metabolic cost as a consequence of the reaction. The mean fitness is the genotype frequency weighted absolute fitness:

[5] $$W_{M,i}=\frac{W_{A(p_{i})}∙n_{\mathrm{pi}}+W_{A(q_{i})}∙n_{\mathrm{qi}}}{M_{i}}.$$

The time derivative for the relative frequency of each genotype is given by the normalized difference between the absolute fitness of the genotype and the mean fitness of the species’ population:

[6] $$\frac{dp_{i}}{\mathrm{dt}}=\frac{W_{A(p_{i})}-W_{M,i}}{W_{M,i}}-m\left(p_{i}\right)+m\left(q_{i}\right),$$

[7] $$\frac{dq_{i}}{\mathrm{dt}}=\frac{W_{A(q_{i})}-W_{M,i}}{W_{M,i}}+m\left(p_{i}\right)-m\left(q_{i}\right),$$

where *m*(*p_i_*) represents the impact of mutation from *p* to *q*, and *m*(*q_i*) represents mutation back in the opposite direction. Mutation is calculated in terms of potential reproductive events that are a function of absolute fitness. The total number of reproductive events for each genotype is the product of the absolute fitness and the relative frequency of the genotype (rounded to the nearest integer):

[8] $${\mathrm{RE}}_{p_{i}}=W_{A\left(p_{i}\right)}∙p_{i},$$

[9] $${\mathrm{RE}}_{q_{i}}=W_{A\left(q_{i}\right)}∙q_{i}.$$

For each reproductive event, a random number between 0 and 1 is generated, from which the mutation parameter *m*_0_ is subtracted. If the result is greater than zero, this event is assumed to correspond to production of the same genotype, if less than zero to the opposite genotype, thus, mutation adjusts the relative frequency of each allele via Eqs. **10** and **11** at each timestep:

[10] $$m\left(p_{i}\right)=\frac{{\mathrm{RE}}_{p_{i}}-\sum_{k=1}^{{\mathrm{RE}}_{p_{i}}}x_{k}}{{\mathrm{RE}}_{p_{i}}},$$

[11] $$m\left(q_{i}\right)=\frac{{\mathrm{RE}}_{q_{i}}-\sum_{k=1}^{{\mathrm{RE}}_{q_{i}}}x_{k}}{{\mathrm{RE}}_{q_{i}}},$$

[12] $$\begin{array}{c} IF\left[\left(RAND\left[0,1\right]-{m_{0}}_{k}\right)>0\right]:x_{k}=1\\ IF\left[\left(RAND\left[0,1\right]-{m_{0}}_{k}\right)\leq 0\right]:x_{k}=0 \end{array},$$

where ${m_{0}}_{k}$ is the baseline mutation rate for the *k* th genotype (i.e., *k* = *p*, *q*), representing the probability that a reproductive event will result in an offspring of the other genotype. Because the capacity to perform the relevant reaction is, by hypothesis, a gain of function phenotype that mutation is more likely to disrupt than create, we make mutation to the wild-type from the reaction performing genotype an order of magnitude more probable than the reverse reaction:

[13] $$\begin{array}{c} {m_{0}}_{p}=m_{0}\\ {m_{0}}_{q}={m_{0}}^{2} \end{array}$$

### The “J-Cycle”.

The relationship between reactions and *J*-variants is depicted in Fig. 1. Variant *J*_1_ is the substrate for reactions *R_A_* and *R_C_*, and the product of reaction *R_B_*. Variant *J*_2_ is the substrate for reaction *R_B_* and the product of reaction *R_D_*. Variant *J*_3_ is the substrate for reaction *R_D_* and the product of reaction *R_C_*. Additionally, each variant is subject to constant abiotic influx *F_in_* and a level-dependent abiotic outflux *F_out_*. Thus, differential equations for the three *J*-variants are given by:

[14] $$\frac{dJ_{1}}{\mathrm{dt}}=F_{\mathrm{in}}-J_{1}∙F_{\mathrm{out}}-R_{A}+R_{B}∙(1-\varepsilon_{X})-R_{C},$$

[15] $$\frac{dJ_{2}}{\mathrm{dt}}=F_{\mathrm{in}}-J_{2}∙F_{\mathrm{out}}+R_{A}∙(1-\varepsilon_{X})-R_{B}+R_{D},$$

[16] $$\frac{dJ_{3}}{\mathrm{dt}}=F_{\mathrm{in}}-J_{3}∙F_{\mathrm{out}}+R_{C}-R_{D},$$

where $\varepsilon_{X}$ is the fraction of element *J* moved into the *X*-cycle as a byproduct of reactions *R_A_* and *R_B_* (see below). Each reaction has the form of an idealized Michaelis–Menten kinetics function. The biological population is assumed to increase the effective substrate availability by a factor of $0<\bar{r_{n}}<1$ per individual, such that when the total number of individuals of the *p* genotype in the relevant species (*i* = *A*, *B*, *C*, *D*) exceeds a threshold $n_{\mathrm{pi}}\geq \frac{1}{\bar{r_{n}}}$, the system is analogous to a set of kinetically limited chemical reactions. This relative biological enhancement of reaction-available *J*-variant is given by

[17] $${\mathrm{BE}}_{i}=MIN[\omega ,n_{\mathrm{pi}}∙\bar{r_{n}}],$$

where 0<ω≤1 is the maximum fraction of the *J*-variant pool that can be made available for the reaction per timestep, by an entire biological population.

[18] $$R_{i}=\frac{R_{\max}∙J_{\mathrm{substrate}}∙{\mathrm{BE}}_{i}}{K_{m}+J_{\mathrm{substrate}}∙{\mathrm{BE}}_{i}},$$

where *R_i_* is in units of substrate *J*-variant converted to product per unit time, *R_max_* is the maximum reaction rate, and *K_m_* is the total effective substrate *J*-variant level at which $R_{i}=\frac{1}{2}R_{\max}$. The cycling ratio of each *J*-variant is given by its level divided by the influx from the outside:

[19] $${\mathrm{CR}}_{x}=\frac{J_{x}}{J_{i0}},$$

where again *x* = 1, 2, 3 as appropriate. The relative abundance of the two cycle biota variants is measured by the geometric mean of the product of the cycling ratio of each substrate *J*-variant and the *p*-genotype driving its reaction through the cycle:

[20] $${\mathrm{CBV}}_{\mathrm{AB}}=\sqrt[{2}]{{\mathrm{CR}}_{1}∙p_{A}∙{\mathrm{CR}}_{2}∙p_{B}},$$

[21] $${\mathrm{CBV}}_{\mathrm{CDB}}=\sqrt[{3}]{{\mathrm{CR}}_{1}∙p_{C}∙{\mathrm{CR}}_{3}∙p_{D}∙{\mathrm{CR}}_{2}∙p_{B}}.$$

The normalized values of CBV relative abundance are:

[22] $$\bar{{\mathrm{CBV}}_{\mathrm{AB}}}=\frac{{\mathrm{CBV}}_{\mathrm{AB}}}{{\mathrm{CBV}}_{\mathrm{AB}}+{\mathrm{CBV}}_{\mathrm{CDB}}},$$

[23] $$\bar{{\mathrm{CBV}}_{\mathrm{CDB}}}=\frac{{\mathrm{CBV}}_{\mathrm{CDB}}}{{\mathrm{CBV}}_{\mathrm{AB}}+{\mathrm{CBV}}_{\mathrm{CDB}}}.$$

### The *X*-Cycle.

Reactions *R_A_* and *R_B_* respectively produce byproduct substances *X_A_* and *X_B_*, at conversion efficiency $\varepsilon_{X}$. These substances exhibit dynamics that are subject to a level-dependent removal flux $X_{out0}$ and react together via additional flux $X_{\mathrm{var}}$:

[24] $$\frac{dX_{A}}{\mathrm{dt}}=\varepsilon_{X}∙R_{A}-X_{out0}∙X_{A}-X_{\mathrm{var}},$$

[25] $$\frac{dX_{B}}{\mathrm{dt}}=\varepsilon_{X}∙R_{B}-X_{out0}∙X_{B}-X_{\mathrm{var}},$$

[26] $$X_{\mathrm{var}}=X_{var0}∙\frac{R_{\max}∙X_{A}∙X_{B}}{K_{m}+X_{A}∙X_{B}}.$$

It is assumed that stoichiometrically one unit of substance *X_A_* and one of substance *X_B_* react together to produce one unit of substance *X*, and $0<X_{var0}<1$ is the baseline efficiency of this reaction. The *X*-cycle is subject to discontinuities derived from inherent feedback processes within it: An initially environmentally unavailable component of the *X*-cycle, $X_{\mathrm{res}}$, analogous to a solid-state subreservoir reacts with the $X_{\mathrm{var}}$ flux, but is not released into the wider environment until the entire initial $X_{\mathrm{res}}$ pool has been depleted from its starting value $X_{res0}$, after which it is released at rate $X_{rel0}$. This is represented by an intermediate “transition pool” $X_{res2}$:

[27] $$\frac{dX_{\mathrm{res}}}{\mathrm{dt}}=-X_{\mathrm{var}}∙X_{\mathrm{res}},$$

[28] $$\frac{dX_{res2}}{\mathrm{dt}}=-\frac{dX_{\mathrm{res}}}{\mathrm{dt}}-X_{\mathrm{release}},$$

[29] $$\begin{array}{c} IF\left[X_{\mathrm{res}}>0\right]:X_{\mathrm{release}}=0\\ IF\left[X_{\mathrm{res}}=0\right]:X_{\mathrm{release}}=X_{rel0}∙X_{res2}+X_{\mathrm{var}} \end{array}.$$

The *X*-cycle is also subject to an inherent bistability, whereby a feedback input occurs between $X_{\mathrm{on}}\leq X\leq X_{\mathrm{off}}$:

[30] $$X_{\mathrm{feedback}}=X_{feedback0}∙\frac{1}{1+e^{-a\left(X-X_{\mathrm{on}}\right)}}∙\frac{1}{1+e^{b\left(X-X_{\mathrm{off}}\right)}}.$$

The overall time derivative for the *X*-cycle is given by:

[31] $$\frac{\mathrm{dX}}{\mathrm{dt}}=X_{\mathrm{in}}-X_{\mathrm{out}}+X_{\mathrm{release}}+X_{\mathrm{feedback}},$$

where $X_{\mathrm{in}}=X_{in0}$ and $X_{\mathrm{out}}=X_{out0}∙X$.

### Cycle Coupling via Suppression Function α(X).

Suppression of species *S_C_* occurs when *X* exceeds a prescribed value $X_{\mathrm{suppress}}$, above which the suppression variable *α* becomes nonzero:

[32] $$IF\left[X< <X_{\mathrm{suppress}}\right]:\;\alpha =0,\;IF\left[X\geq X_{\mathrm{suppress}}\right]:\;\alpha =\frac{\alpha_{0}}{1+e^{-k_{\alpha }(X-X_{\mathrm{suppress}})}},$$

where $k_{\alpha }=20$ is a smoothing term to prevent instabilities arising from discontinuous jumps in *α*. The time derivative for the population of *S_C_* is modified from that given in Eq. **2** to incorporate a per capita “kill” effect:

[33] $$\frac{dM_{C}}{\mathrm{dt}}\to \frac{dM_{C}}{\mathrm{dt}}-M_{C}∙\alpha .$$

### Numerical Solution by the Euler–Maruyama Method.

The overall dynamics of species biomass (Eq. **2**) were subject to a stochastic effect dictated by relative fitness differences. The dynamics of the *J*-cycle were subject to a stochastic effect dictated by biomass fluctuations and their impact on reaction rates. Finally, the *X* cycle dynamics were subject to a stochastic effect dictated by the impact of species abundance on the above-mentioned byproducts. In each case, the final derivative for the system was of the form:

[34] $$\frac{dy_{\mathrm{system}}}{\mathrm{dt}}=f_{\mathrm{system}}\left(y,t\right)dt_{\mathrm{system}}+\bar{\sigma }g_{\mathrm{system}}\left(y,t\right)\sqrt{dt_{\mathrm{system}}}∙N(0,1),$$

where “*system*” denotes “*bio*,” “*J*” or “*X*” as appropriate, *f_system_* denotes the deterministic part of the derivative (given above), *g_system_* denotes the diffusion term, N(0,1) is a standard normal random variable, and $\bar{\sigma }$ is a parameter dictating the overall sensitivity of the system to the stochastic components of the model. (Qualitative model features are retained by setting $\bar{\sigma }=0$ and removing this stochastic component).

For the biological dynamics, the diffusion term was a function of the variance in allele frequencies, given by the binomial distribution describing the expected number of mutations:

[35] $$g_{S_{i}}=\sqrt{\frac{m_{0,p_{i}}∙(1-m_{0p_{i}})}{{\mathrm{RE}}_{p_{i}}}∙\frac{m_{0,q_{i}}∙(1-m_{0q_{i}})}{{\mathrm{RE}}_{q_{i}}}},$$

[36] $$dM_{i}=\frac{dM_{i}}{\mathrm{dt}}∙{\mathrm{dt}}_{\mathrm{bio}}+\bar{\sigma }∙g_{S_{i}}∙N(0,1)∙\sqrt{{\mathrm{dt}}_{\mathrm{bio}}}.$$

The diffusion terms for each *J*-variable were calculated as a function of the sum of squares for each reaction appearing in the deterministic differential equation for that variable:

[37] $$g\left(J_{x}\right)=\sqrt{\sum_{k}{g_{R_{i},k}}^{2}},$$

where each reaction-specific term $g_{R_{i}}$ is the product of the partial derivative of the reaction rate with respect to the number $n_{\mathrm{pi}}$ of reaction-performing individuals of the relevant genotype, and the SD of the number of individuals of that genotype:

[38] $$g_{R_{i}}=\frac{\partial R_{i}}{\partial n_{\mathrm{pi}}}∙\sqrt{VAR\left(n_{\mathrm{pi}}\right)},$$

where:

[39] $$\frac{\partial R_{i}}{\partial n_{\mathrm{pi}}}=\omega ∙\frac{K_{m}∙R_{\max}∙J_{\mathrm{substrate}}∙n_{\mathrm{pi}}∙\bar{r_{n}}}{{\left(K_{m}+J_{\mathrm{substrate}}∙n_{\mathrm{pi}}∙\bar{r_{n}}\right)}^{2}},$$

and where the variance in the number of individuals was given by:

[40] $$VAR\left(n_{\mathrm{pi}}\right)=VAR\left(p_{i}∙S_{i}\right)={p_{i}}^{2}∙VAR\left(S_{i}\right)+{S_{i}}^{2}∙VAR\left(p_{i}\right).$$

With the variance in the reaction-performing allele frequency comparable to (Eq. **35**) above, but without the wild-type component of the calculation:

[41] $$VAR\left(p_{i}\right)=\sqrt{\frac{m_{0,p_{i}}∙(1-m_{0p_{i}})}{{\mathrm{RE}}_{p_{i}}}}.$$

The variance in the total number of individuals $VAR\left(S_{i}\right)$ was assumed to be approximated by:

[42] $$VAR\left(S_{i}\right)=W_{A\left(p_{i}\right)}∙p_{i}∙\left(1-p_{i}\right)+W_{A\left(q_{i}\right)}∙q_{i}∙\left(1-q_{i}\right).$$

Plugging each of these terms in gave the final derivative form for the *J*-cycle:

[43] $$dJ_{x}=\frac{dJ_{x}}{\mathrm{dt}}∙{\mathrm{dt}}_{J}+\bar{\sigma }∙g\left(J_{x}\right)∙N(0,1)∙\sqrt{{\mathrm{dt}}_{J}}.$$

For *x* = 1, 2, 3 as relevant.

The stochastic diffusion terms within the byproduct dynamics of the X cycle were of the same form as those above, with (Eq. **24**) modified by inclusion of a term of the form:

[44] $$g_{X_{i}}=\frac{\partial R_{i}}{\partial n_{\mathrm{pi}}}∙\sqrt{VAR\left(n_{\mathrm{pi}}\right)},$$

where i = *A*, *B*, and using (Eqs. **39** and **40**). Thus:

[45] $$dX_{i}=\frac{dX_{i}}{\mathrm{dt}}∙dt_{X}+\bar{\sigma }∙g_{X_{i}}∙N(0,1)∙\sqrt{{\mathrm{dt}}_{X}}.$$

The overall diffusion term for the main *X* pool $g_{X_{\mathrm{main}}}$ incorporated the potential for stochastic effects being propagated by the biologically influenced fluxes (the other parts of this cycle being assumed to be deterministic):

[46] $$g_{X_{\mathrm{main}}}=\sqrt{{g_{X_{A}}}^{2}+{g_{X_{B}}}^{2}+{\frac{\partial X_{\mathrm{var}}}{\partial X_{A}}}^{2}∙{g_{X_{A}}}^{2}+{\frac{\partial X_{\mathrm{var}}}{\partial X_{B}}}^{2}∙{g_{X_{B}}}^{2}}.$$

Using (Eq. **44**). Equivalently, using (Eq. **26**):

[47] $$\begin{array}{c} \frac{\partial X_{\mathrm{var}}}{\partial X_{A}}=\frac{R_{\max}∙X_{B}∙\left(K_{m}+X_{A}∙X_{B}\right)-X_{B}∙(R_{\max}∙X_{A}∙X_{B})}{(K_{m}+X_{A}∙X_{B}{)}^{2}}\\ \;=\frac{R_{\max}∙X_{B}∙K_{m}}{(K_{m}+X_{A}∙X_{B}{)}^{2}}, \end{array}$$

[48] $$\frac{\partial X_{\mathrm{var}}}{\partial X_{B}}=\frac{R_{\max}∙X_{A}∙K_{m}}{{\left(K_{m}+X_{A}∙X_{B}\right)}^{2}}.$$

Thus, the final derivative for the *X*-cycle was of the form:

[49] $$dX=\frac{\mathrm{dX}}{\mathrm{dt}}∙dt_{X}+\bar{\sigma }∙g_{X_{\mathrm{main}}}∙N(0,1)∙\sqrt{{\mathrm{dt}}_{X}}.$$

The deterministic derivatives were assumed to be subject to the partial timescale separation ${\mathrm{dt}}_{\mathrm{bio}}<dt_{J}<dt_{X}$, with $dt={\mathrm{dt}}_{\mathrm{bio}}$ for (Eq. **2**), (Eq. **6**) and (Eq. **7**) and (Eq. **33**), $dt=dt_{J}$ for (Eqs. **14**–**16**), and $dt=dt_{X}$ for (Eqs. **24**–**31**). The relative magnitude of these timesteps was parameterized in terms of $\tau_{J}>1$ and $\tau_{X}>1$, i.e:

[50] $${\mathrm{dt}}_{\mathrm{bio}}∙\tau_{J}∙\tau_{X}={\mathrm{dt}}_{J}∙\tau_{X}={\mathrm{dt}}_{X}.$$

However, in order to avoid errors arising from numerical instabilities while investigating the impact of the timescale separation as a parameter, each set of dynamics was numerically integrated at each biological timestep, meaning that the longer timescale dynamics of the *J*-cycle and *X*-cycle were respectively scaled by a factor of $\frac{1}{\tau_{J}}$ and $\frac{1}{\tau_{J}∙\tau_{X}}$ for each (integer) unit of ${\mathrm{dt}}_{\mathrm{bio}}$.

Default parameter choices are given in Table 1. The full set of stochastic differential equations was solved in MATLAB using the Euler–Maruyama method. Full MATLAB code is available at https://github.com/richboyle111/ITSNTS_new. For sensitivity analyses, parameters not being investigated were held at default values unless otherwise stated.

## Supplementary Material

Appendix 01 (PDF)

## Data Availability

There are no data underlying this work.
