# Supplementary material for: Persistence selection between simulated biogeochemical cycle variants for their distinct effects on the Earth system
Source: Proc Natl Acad Sci U S A. 2025 Feb 12;122(7):e2406344122. doi: 10.1073/pnas.2406344122 (PMC11848429; doi:10.1073/pnas.2406344122)
Supplement: Supplementary file 1 — Appendix 01 (PDF) [file pnas.2406344122.sapp.pdf]

Supplementary Information:

Quasi-Darwinian persistence-selection between simulated biogeochemical cycle-variants for their distinct effects on the Earth-system

Supplementary methods S1

Model 2: Derivation of probability estimates discussed in main text

Consider the state of an environmental context E_L, which can be labelled "local" in the sense that it exhibits dynamics over similar spatial and temporal scales to evolutionary ecology. Suppose that E_L is affected by a total of N different species within the biosphere, via relationships of the form f_E:

$$E_L = E_{L,abiotic} + \sum_{i=1}^{N} f_E(g_i) \quad (2.1)$$

Where $E_{L,abiotic}$ is the state that E_L would have in the absence of life, $f_E(g_i)$ is the impact of species i on E_L. Assume that the state of the environment can be discretized such that E_L occupies only one of a finite set of L possible integer values. Assume that the baseline abiotic state is the median value of a uniform distribution, meaning that the range of states that E_L may exhibit is constrained within:

$$E_L \in U(1,L) |\, L \neq \infty, \forall t, E_{L,abiotic} = Med\big(U(1,L)\big) = L/2 \quad (2.2)$$

Assume that species affect E_L through some impact phenotype x_i, the effect of which is to cause E_L to jump to one of the other possible values:

$$f_E(g_i) = x_i \mid E_L(t) + x_i = U(1,L)\ \forall i \quad (2.3)$$

Assume that the value of x_i is random with respect to organism evolution, not an adaptation that will benefit the genotype, meaning that any given x_i is a randomly generated integer in the range $\big(-E_{L,abiotic}, E_{L,abiotic}\big)$. Make the simplifying assumption that edge effects and the overall question of collective habitability can be ignored for the purposes of the focus here, such that if the biosphere pushes E_L out of the viable range of values, the system simply jumps back to the lower end of the distribution:

$$IF\ (E_L + x_i > L):\ E_L \rightarrow L - (E_L + x_i) \quad (2.4)$$

In keeping with the main model, assume that the impact phenotype is exhibited by only $g_{i,E}$, one of two genotypes within each of the N species, not the wildtype genotype $g_{i,0}$:

$$g_{i,E} + g_{i,0} = 1,\ f_E\big(g_{i,E}\big) = x_i,\ f_E\big(g_{i,0}\big) = 0, \forall i = 1,2 \ldots N \quad (2.5)$$

Let the fitness of the environment-affecting allele differ from that of the wild type by a factor of $-1 < s_{g_{i,E}} < 1$. Let the fitness of both genotypes be a function of a genotype-specific optimum state of E_L, $E_{L,opt,k}$ (for $k = 0, E$):

$$W_{g_{i,E}} = \big(1 + s_{g_{i,E}}\big) \cdot f_{bio,i,E}(E_L) \quad (2.6)$$

$$W_{g_{i,0}} = f_{bio,i,0}(E_L) \quad (2.7)$$

$$IF\big(E_L = E_{L,opt,k}\big):\ f_{bio,k} = f_{bio,max},\ ELSE: f_{bio,k} = f_{bio,min},\ \forall k \quad (2.8)$$

Where the parameters $0 < f_{bio,max} \leq 1$ and $0 < f_{bio,min} \leq 1$, $f_{bio,min} < f_{bio,max}$ define the magnitude of the impact of E_L on fitness.

The relative impact contribution $E_{L,i}$ to the overall state of E_L is equivalent to the value that E_L would take if that species were the only one present:

$$E_{L,i} = E_{L,abiotic} + x_i, ELSE: E_{L,i} = 0, \forall i \quad (2.9)$$

Assume an additional constraint that the nature of physiological byproducts is such as to preclude any single genotype from inducing its own optimal state. (For instance, aerobic organisms require oxygen, but impact upon their environments by consume it. Thus, aerobic organisms do not directly promote the high oxygen conditions from which they benefit but can do so only in conjunction with oxygenic photosynthesizers. Assume that this idea of reciprocity holds generally). Thus:

$$E_{L,i} \neq E_{L,opt,i}, \ \forall i \quad (2.10)$$

Define an E_L-based reciprocal "symbiotic" interaction $I_{u,v}$ as a scenario in which the environment-affecting allele has reached fixation in two species u and v, and the impact of each species is to cause E_L to jump to the optimum value for the other. Exclude the possibility that $E_{L,opt,u} = E_{L,opt,v}$ because this would amount to fortuitous convergence of optima rather than reciprocal interactive regulation. Assume that such an interaction is stable if and only if (IFF) the environment-affecting alleles in each species have reached fixation. Thus, manifestation of an interaction of this sort is definable as:

$$\exists I_{u,v} \ IFF \begin{bmatrix} \left((g_{i,u} = 1) \ AND \ (g_{i,v} = 1)\right) \\ AND \ \left((E_{L,opt,u} - x_v = E_{L,opt,v} - x_u) \middle| E_{L,opt,u} \neq E_{L,opt,v}\right) \\ AND \ \left((E_L = E_{L,opt,u}) \ OR \ (E_L = E_{L,opt,v}) \right) \end{bmatrix} \quad (2.11)$$

Importantly, note that this implies that the simultaneous presence of multiple such interactions with non-overlapping optima is likely unstable, due to the mutual disruption of optimal conditions, whereby (say) species u and v undermines the stable cyclic effect resulting from the interaction between species q and p, by pulling the system away from the overlapping optima for the latter.

Suppose that Darwinian dynamics are such that, given an interaction of the form of (2.11), it is only a matter of time before such competitive dynamics emerge, either at a meta-interaction level, or in terms of evolutionary innovations that undermine the local fixation of the required environment affecting alleles. Define, with respect to the interaction $I_{u,v}$, a "Darwinian decay time" describing the likelihood that this will occur:

$$t_{decay}(I_{u,v}) = t_{decay0} \cdot \left(1 + \sum_{i=1}^{N} (g_{i,E})_{i \neq u.v}\right) \quad (2.12)$$

Where t_{decay0} is a parameter, and the summation term gives the number of other fixed environment-affecting alleles in the population, which will dictate the probability of competitive "meta-interactions".

Define a second environmental context E_G, which is "global" in nature in the sense that it adjusts over qualitatively greater spatial and temporal scales than those of the Darwinian dynamics. Suppose that it exhibits equivalent properties to the local environmental context, except that it is potentially affected by interaction-level byproducts, rather than by individual species:

$$E_G \in \ U(1, G) | \ G \neq \infty, \forall t, E_{G,abiotic} = Med\left(U(1, G)\right) \quad (2.13)$$

$$E_G = E_{G,abiotic} + \sum_{j=1}^{M} f_E(I_j) \quad (2.14)$$

Where G is the number of possible (integer) states that E_G can exhibit, M is the number of interactions of the form of (11) that are present, each of which affects E_G via a relationship of the form f_E, and again the baseline abiotic state is just the median of the permitted rand $E_{G,abiotic} = G/2$ (or the nearest integer). Suppose that any impact "phenotype" (i.e. global environmental effect of an

interaction of the form of (11)) can only manifest after a parameterized lag time t_{lag} has elapsed (representing the relevant separation of spatial and temporal scales), and only if competitive meta-interaction competition has not emerged within this time. Represent the probability that the latter occurs as a function of the ratio between this lag time and t_{decay}, to give a condition for $I_{u,v}$ having an impact $y_{I_{u,v}}$ on E_G:

$$IF\left(t_{lag} < t_{decay}\right),\ f_E\left(I_j\right) = y_{I_j} > 0\ , ELSE\colon y_j = 0, \forall j \tag{2.15}$$

Assume that if this condition is realized, y is a randomly generated integer in the range $\left(-E_{G,abiotic}, E_{G,abiotic}\right)$, equivalent to x above but applying to the global environmental context. Equivalent to (4), make the simplifying assumption that E_G jumps back to the other end of the distribution if pushed outside the range of permitted states:

$$IF\ (E_G + y_i > G)\colon E_G = G - (E_G + y_i) \tag{2.16}$$

Crucially, suppose that if an interaction occurs of the form of $I_{u,v}$ does persist long enough to influence E_G, the effect of this influence may feed back onto the more local scales and alter the sort of interactions that are feasible. (For example, the evolution of oxygenic photosynthesis had wider climatic effects that ultimately resulted in a geochemical bi-stability, leading to the spread and stabilization of aerobic niches at ecological spatial and temporal scales). Suppose that only one possible value for the state of the global environmental context E_G is optimal for the persistence of this interaction, in the sense that it is a necessary and sufficient condition for this interaction to "kill off" all competitor interactions, analogously to the suppression effect illustrated by our dynamical model. Label this scenario as:

$$E_{G,opt,(I_j)} = E_G + y_{I_j} \tag{2.17}$$

This scheme allows us to summarize the constituent individual events necessary and sufficient for the sort of general trajectory we propose (main text). The "fractional" result shown in figure S1 derives from the random generation of populations of species, and interaction traits conforming to equations (1-17) by straightforward sampling from the relevant uniform distributions.

Additionally, we derive an analytic approximation to the probability that each of the above conditions are met. Assuming local environmental context E_L is initially equally likely to exhibit any of the L possible state values, and that the optimal environmental state value for each allele is selected at random from this same set of possibilities, then the expected value of the fitness terms for the wild type and environment-affecting alleles are, respectively:

$$E\left(W_{g_{i,0}}, E_L\right) = \frac{f_{bio,max}}{L} + \left(\frac{L-1}{L}\right) f_{bio,min} \tag{2.18}$$

$$E\left(W_{g_{i,E}}, E_L\right) = \left(\frac{f_{bio,max}}{L} + \left(\frac{L-1}{L}\right) f_{bio,min}\right) \cdot \left(1 + s_{g_{i,E}}\right) \tag{2.19}$$

Kimura's formula for the fixation probability for the environment affecting allele is, under conventional population-genetic assumptions, of the form $\frac{1-e^{-2spN_e}}{1-e^{-2N_e s}}$, where s is the selection-coefficient, N_e is the effective population size, and p is the allele frequency. Assume that the focal allele begins to increase from rarity at a very low frequency, so that initially $p \approx \frac{1}{N_e}$. Given the above expected values for the fitness terms, this selection coefficient can be approximated by $s = \frac{E\left(W_{g_{i,E}}, E_L\right) - E\left(W_{g_{i,0}}, E_L\right)}{E\left(W_{g_{i,0}}, E_L\right)} = s_{g_{i,E}}$. Thus, this fixation probability for the environment affecting allele is:

$$P_{fix}, g_{i,E} = \frac{1-e^{-2s_{g_{i,E}}}}{1-e^{-2N_{e,i}s_{g_{i,E}}}} \tag{2.20}$$

With respect to the interaction condition $E_{L,opt,u} - x_v = E_{L,opt,v} - x_u$, there are L^4 possible combinations of values for these independently varying parameters. There are therefore $L \cdot (L-1)$ pairings for the two optima terms, each of which is associated with a single unique combination of the two x terms in order to satisfy the condition. Thus, the probability that this interaction condition will be realized is $\frac{L \cdot (L-1)}{L^4}$:

$$P(E_{L,opt,u} - x_v = E_{L,opt,v} - x_u) = \frac{L-1}{L^3} \quad (2.21)$$

The probability of realization of the final condition in (11), i.e. that the initial environmental state matches one of the optima is $\frac{2}{L}$ (the possibility that $E_{L,opt,u} = E_{L,opt,v}$ having already been excluded). giving the final probability for a symbiotic interaction:

$$P(I_{u,v}) = \left(\frac{1-e^{-2sg_{u,E}}}{1-e^{-2N_{e,u} \cdot s g_{u,E}}}\right) \cdot \left(\frac{1-e^{-2sg_{v,E}}}{1-e^{-2N_{e,v} \cdot s g_{v,E}}}\right) \cdot \left(\frac{2(L-1)}{L^4}\right) \quad (2.22)$$

The "Darwinian decay time" described by (12) is a function of the expected number of other environment-affecting alleles that will be fixed, other than those in the initial interaction-forming species u and v. Approximate any one of these fixation-probabilities by a population-averaged version of (20):

$$P_{fix,g_{average,E}} = \frac{1-e^{-2sg_{average,E}}}{1-e^{-2N_{e,average} s g_{average,E}}} \quad (2.24)$$

Then, inserting (2.12) into (2.15), the "Darwinian decay time" condition can be written as a relationship between the ratio of the two key timescales, and the number of other environment-affecting alleles that have been fixed:

$$\frac{t_{lag}}{t_{decay0}} < \left(1 + \sum_{i=1}^{N} (g_{i,E})_{i \neq u.v}\right) = 1 + S \quad (2.25)$$

Where the probability that the focal interaction will survive local "Darwinian disruption" is $S < S_{crit}$, where:

$$S_{crit} = \frac{t_{lag}}{t_{decay0}} - 1 \quad (2.26)$$

Using (2.24) we can approximate this probability with a binomial distribution:

$$P(t_{lag} < t_{decay}) = 1 - \sum_{k=0}^{S_{crit}} \binom{N-2}{k} \cdot P_{fix,g_{average,E}}{}^{k} \cdot (1 - P_{fix,g_{average,E}})^{N-2-k} \quad (2.27)$$

Finally, in terms of the meta-interaction competition condition (2.18), by hypothesis, the global environmental context E_G is not yet "optimal" for the focal interaction $I_{u,v}$, but a long-term side effect may cause it to become so. This implies $E_{G,opt,(I_j)} \neq E_G$, which in turn means that there are $2G - 2$ possible values for $E_{G,opt,(I_j)} - E_G$, for which only one y_{I_j} value will satisfy the condition. If there exist G possible values for y_{I_j}, the final probability of this occurring is:

$$P\left(E_{G,opt,(I_j)} = E_G + y_{I_j}\right) = \frac{G}{2G-2} \quad (2.28)$$

Thus, the overall probability that an environmentally influential "symbiotic" interaction will arise, persist long enough to avoid disruption from local Darwinian dynamics, and then elicit a long-term biogeochemical/climatic effect that causes it to displace competitor interactions of the same form is given by:

$$P_{ITSNTS} = P(I_{u,v}) \cdot P(t_{lag} < t_{decay}) \cdot P\left(E_{G,opt,(I_j)} = E_G + y_{I_j}\right) \quad (2.29)$$

This final probability is the product of (2.22) (which we assume can be approximated by substituting in (2.24)), (2.27), and (2.28). Figure S1 in the main text compares the analytic approximation (2.29) of this probability to a direct sampling process involving generating a population of fitness and environmental interaction terms conforming to equations (1)-(17), figure S2 decomposes this probability into its constituent parts. Differences between the sampling based and analytical estimates result primarily from the latter's neglecting of between species variation in fixation probability.

Figure S1 – Comparative analysis of analytic and sampling-based estimates of the probability of CBV-level persistence selection making a difference to allele frequencies. Each panel shows the probability P_{ITSNTS} as a function of key parameters L, the number of states for the local environmental context, G, the number of states for the global context (row A), $N_{e,average}$, the average effective population size within each species, $s_{g_{average,E}}$, the average selection coefficient experience by the environment affecting allele (row B), N, the total number of species, and the ratio $\frac{t_{lag}}{t_{decay0}}$ between the time taken for a global scale effect and the time for local Darwinian dynamics to disrupt that effect (row C). Column 1 shows an estimate of the fraction of sample space corresponding to the necessary conditions, column 2 shows an analytical approximation to the probability that all these conditions will be simultaneously realized.

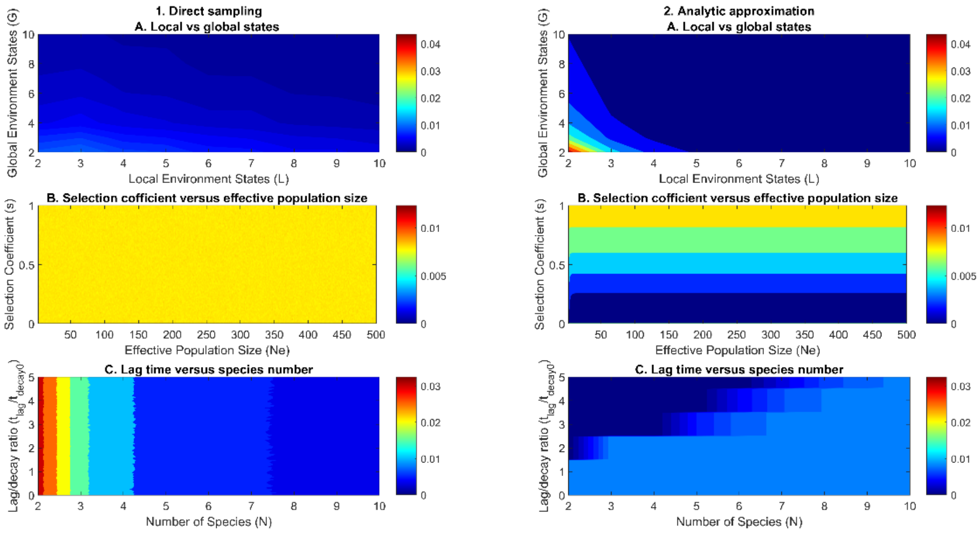

Supplementary Figure S2 – expanded estimate of the factors affecting P_{ITSNTS} and the constituent probabilities

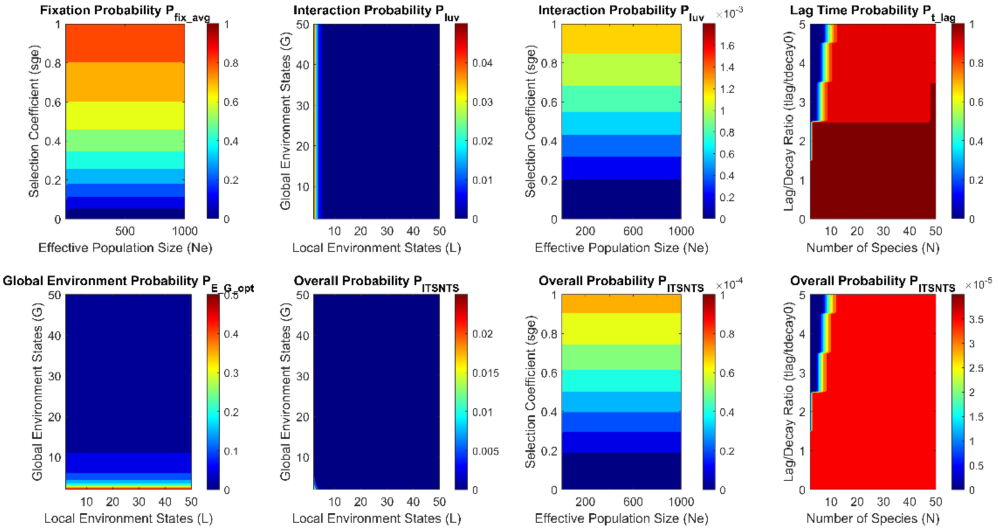

Constituent probabilities of (29), broken down into (24), (27) and (28), as a function of the state diversity of the local and global environmental context, and the Darwinian parameters. “Overall probability” denotes P_{ITSNTS} calculated according to equation (2.29), main text, “Fixation probability” to (2.24), “global environment probability” to (2.28).

Supplementary methods S2:

Phylogenetic reconstruction of metabolic completeness

To estimate metabolic completeness across time, we used the reconciliation data from Moody *et al.* (2024). Individual gene families across each node in the species tree were determined as being ‘present’ where they had a probability of presence equal to or greater than 75%. These present gene families were then used to estimate metabolic completeness using the ‘anvi-estimate-metabolism’ function (pathwise) in Anvi’o v8. Metabolic completeness data was filtered to exclude values of zero (i.e. none of the requisite genes present in a given metabolic pathway). Metabolic pathwise completeness at each node in the species tree, was then plotted against that node’s respective mean age divergence time estimate (see above), and a LOESS regression with ggplot in R.

Following Moody et al. (2024), we constrained the age of eight nodes: LUCA (Last Universal Common Ancestor), Total-Group (TG) Archaea, TG Mitochondria, Crown-Group (CG) Chlamydia, TG Oxyphotobacteria, CG Oxyphotobacteria. The age of node TG Chromatiaceae was constrained following Mahendrarajah et al. 2024, In addition to this, we also constrained the node for TG Nostocales based on (Davin *et al*). The GOE (Great Oxidation Event), was used to calibrate [Thaumarcheota/Nitrososphaerota], and CG Archaea (excluding DPANN) as an additional deep archaeal calibration:

Crown-Archaea excluding DPANN | 3331-4520 Ma

Clade: Total-group of the clade composed of Archaea but not DPANN.

Fossil taxon and specimen: Filaments colonizing the walls of conduits created by low-temperature hydrothermal fluid, within a hydrothermal chert vein system underlying a marine chert (K1c1) located near the base of the Kromberg Formation, Onverwacht Group, Barberton Greenstone Belt, South Africa (1).

Minimum Age justification: The age of the Kromberg Formation has been constrained by Pb-Pb and U-Pb dating of zircons from tuffs near its base and top, dated to 3416 Ma ± 5 Myr and 3334 Ma ± 3 Myr, respectively (2). Since the age relationship between the basal tuff and the fossil bearing chert is unknown, we take the age of the upper tuff to establish the minimum age constraint, thus, 3331 Ma.

Hard maximum age justification: The Moon forming impact would have effectively sterilized the Earth and so it serves as an effective basis for establishing a hard maximum age constraint on LUCA. Pb-Pb dating carried out on Moon rocks, yielding a date of 4.51 Ga ± 10 Myr (3) a date which has also recently been confirmed by reanalysis of the Apollo mission zircons (4). Thus, our maximum constraint is 4.52 Ga.

Discussion: There are claims of methanogens based on isotope data alone, dated to ~ 3.5 Ga (5), but it is not yet possible to preclude the possibility that they represent abiotic reduction of CO2 to CH4 (6). The combined record of filamentous microfossils and geochemical evidence of methanogenesis from the Kromberg Formation, Onverwacht Group, Barberton Greenstone Belt (1), is more credible.

Timetree inference

All our timetree inference analyses were carried out with the dating program MCMCtree (PAML v4.10.7, Yang 2007) under the autocorrelated-rates (or Geometric Brownian motion,

GBM; Thorne et al. 1998, Yang and Rannala 2006) and the Independent-rates Log-Normal (ILN; Rannala and Yang 2007, Lemey et al. 2007) relaxed-clock models. We tested the impact that constraining the age of node CG Archaea (excluding DPANN) could have on timetree inference. Therefore, we ran two analyses under both relaxed-clock models under two calibration strategies: one with the age of such a node was unconstrained and another in which a uniform distribution with soft bounds (minimum age: 3,331 Ma; maximum age: 4,520 Ma) was used as a constraint.

To define our rate prior, we first had to estimate the mean evolutionary rate. To do so, we divided the tree height of the phylogeny inferred by Moody et al. (2022) into the estimated mean root age (i.e., average between the minim and maximum ages used to constrain the root age, that is 3.9335 Ga as our time unit was set to 1 Ga), which resulted in 7.37e-10 substitutions/site/year. To account for the uncertainty in our rate estimate, we used a diffuse gamma rate prior by setting alpha = 2 (shape parameter). We calculated the value of the scale parameter (beta) by dividing the value of alpha into the estimated mean evolutionary rate, and thus our rate prior was $\Gamma(2,2.7)$. Given the depth of our phylogeny, we assume the clock to be violated and estimate a large rate variance, $\sigma^2 = 0.1$. Accordingly, we fitted a vague gamma prior centered around such value with the aim to integrate our uncertainty on such an estimate, $\Gamma(1, 10)$. Lastly, we set a birth-death process with sampling (Yang and Rannala, 1997) with $\lambda = \mu = 1$ (i.e., equal per-lineage birth and death rate, respectively) and $\rho = 0.1$ (sampling fraction); parameter values that yield a uniform kernel density with which equal probabilities are assumed for all possible tree shapes.

We aimed to collect a total of 20,000 samples per chain, but only managed to do so when the target distribution was the prior (i.e., no data were used; we ran a total of 6 independent chains when sampling from the prior). To increase the effective sample size when the target distribution was the posterior (i.e., data are used), we decided to run 16 independent chains under each strategy and relaxed-clock model (i.e., 16x4=64 chains), which allowed us to collect enough samples within the allowed wall time restriction in the HPC we used to properly summarize the posterior.

We carried out all MCMC diagnostics following the same procedure described in Moody et al. (2024), which are also explained in our GitHub repository.

Supplementary Figure S3 – Metabolic completeness of methane metabolism pathways through time. A LOESS regression plot showing the completeness of different KEGG methane metabolism (map00680) modules across time in millions of years.

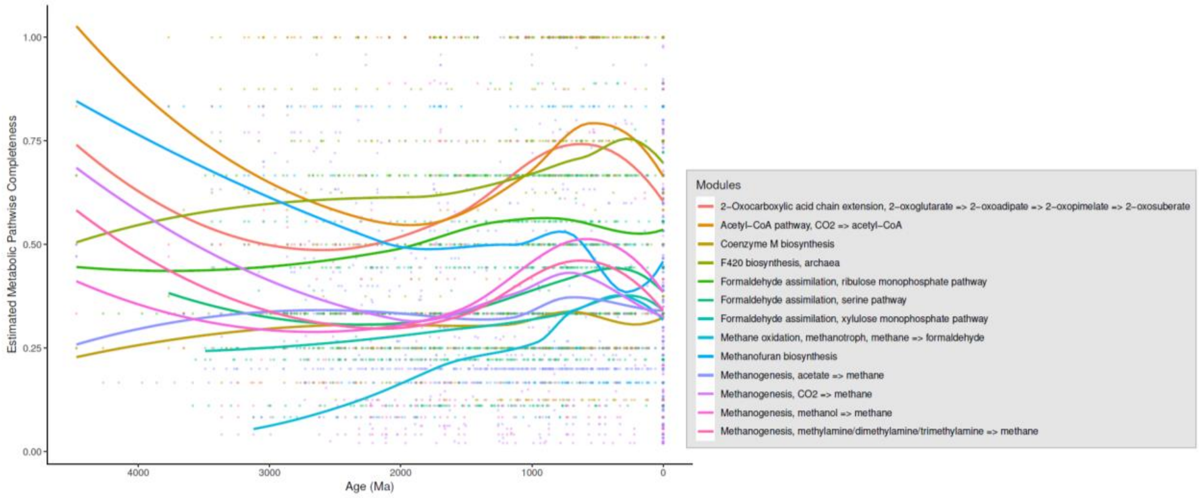

Supplementary Figure S4 – Metabolic completeness of nitrogen metabolism pathways through time. A LOESS regression plot showing the completeness of different KEGG nitrogen metabolism (map00910) modules across time in millions of years.

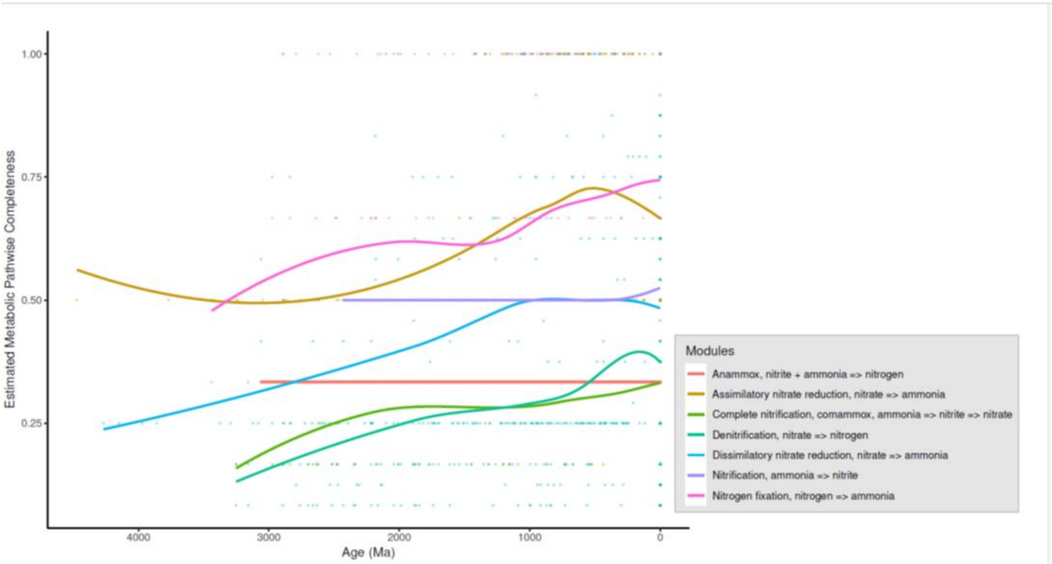

Supplementary References (in order cited):

1. Yang 2007 | Ziheng Yang, PAML 4: Phylogenetic Analysis by Maximum Likelihood, Molecular Biology and Evolution, Volume 24, Issue 8, August 2007, Pages 1586–1591, https://doi.org/10.1093/molbev/msm088.
2. Thorne et al. 1998 | Thorne JL, Kishino H, Painter IS. Estimating the rate of evolution of the rate of molecular evolution. Mol Biol Evol. 1998 Dec;15(12):1647-57. doi: 10.1093/oxfordjournals.molbev.a025892.

3. Yang and Rannala 2006 | Yang Z, Rannala B. Bayesian estimation of species divergence times under a molecular clock using multiple fossil calibrations with soft bounds. Mol Biol Evol. 2006 Jan;23(1):212-26. doi: 10.1093/molbev/msj024.
4. Rannala and Yang 2007 | Rannala B, Yang Z. Inferring speciation times under an episodic molecular clock. Syst Biol. 2007 Jun;56(3):453-66. doi: 10.1080/10635150701420643.
5. Lemey et al. 2007 | Lemey P, Rambaut A, Welch JJ, Suchard MA. Phylogeography takes a relaxed random walk in continuous space and time. Mol Biol Evol. 2010 Aug;27(8):1877-85. doi: 10.1093/molbev/msq067.
6. Moody et al. (2022) | Moody ERR, Mahendrarajah TA, Dombrowski N, Clark JW, Petitjean C, Offre P, Szöllősi GJ, Spang A, Williams TA. An estimate of the deepest branches of the tree of life from ancient vertically evolving genes. Elife. 2022 Feb 22;11:e66695. doi: 10.7554/eLife.66695.
7. Yang and Rannala, 1997 | Yang Z, Rannala B. Bayesian phylogenetic inference using DNA sequences: a Markov Chain Monte Carlo Method. Mol Biol Evol. 1997 Jul;14(7):717-24. doi: 10.1093/oxfordjournals.molbev.a025811.
8. Moody et al. 2024 | Moody, E.R.R., Álvarez-Carretero, S., Mahendrarajah, T.A. *et al.* The nature of the last universal common ancestor and its impact on the early Earth system. *Nat Ecol Evol* **8**, 1654–1666 (2024). https://doi.org/10.1038/s41559-024-02461-1
9. B. Cavalazzi *et al.*, Cellular remains in a~ 3.42-billion-year-old subseafloor hydrothermal environment. *Science Advances* **7**, eabf3963 (2021).
10. Byerly, A. Kröner, D. R. Lowe, W. Todt, M. M. Walsh, Prolonged magmatism and time constraints for sediment deposition in the early Archean Barberton greenstone belt: evidence from the Upper Onverwacht and Fig Tree groups. *Precambrian Research* **78**, 125-138 (1996).
11. B. Hanan, G. Tilton, 60025: relict of primitive lunar crust? *Earth and Planetary Science Letters* **84**, 15-21 (1987).
12. M. Barboni *et al.*, Early formation of the Moon 4.51 billion years ago. *Science advances* **3**, e1602365 (2017).
13. Y. Ueno, K. Yamada, N. Yoshida, S. Maruyama, Y. Isozaki, Evidence from fluid inclusions for microbial methanogenesis in the early Archaean era. *Nature* **440**, 516-519 (2006).
14. K. Lepot, Signatures of early microbial life from the Archean (4 to 2.5 Ga) eon. *Earth-Science Reviews* **209** (2020).
15. Mahendrarajah TA, Moody ERR, Schrempf D, Szánthó LL, Dombrowski N, Davín AA, Pisani D, Donoghue PCJ, Szöllősi GJ, Williams TA, Spang A. ATP synthase evolution on a cross-braced dated tree of life. Nat Commun. 2023 Nov 17;14(1):7456. doi: 10.1038/s41467-023-42924-w.
16. Adrián A. Davín, Ben J. Woodcroft, Rochelle M. Soo, Benoit Morel, Ranjani Murali, Dominik Schrempf, James Clark, Bastien Boussau, Edmund R. R. Moody, Lénárd L. Szánthó, Etienne Richy, Davide Pisani, James Hemp, Woodward Fischer, Philip C.J. Donoghue, Anja Spang, Philip Hugenholtz, Tom A. Williams, Gergely J. Szöllősi, An evolutionary timescale for Bacteria calibrated using the Great Oxidation Event. BioRxiv doi: https://doi.org/10.1101/2023.08.08.552427

17. Moody, E.R.R., Álvarez-Carretero, S., Mahendrarajah, T.A. *et al.* The nature of the last universal common ancestor and its impact on the early Earth system. *Nat Ecol Evol* **8**, 1654–1666 (2024). https://doi.org/10.1038/s41559-024-02461-1
18. Eren, A.M., Kiefl, E., Shaiber, A. *et al.* Community-led, integrated, reproducible multi-omics with anvi'o. *Nat Microbiol* **6**, 3–6 (2021). https://doi.org/10.1038/s41564-020-00834-3
19. Wickham H (2016). ggplot2: Elegant Graphics for Data Analysis. Springer-Verlag New York. ISBN 978-3-319-24277-4, https://ggplot2.tidyverse.org
20. R Core Team (2021). R: A language and environment for statistical computing. R Foundation for Statistical Computing, Vienna, Austria. URL https://www.R-project.org/.
